# A Pilot Study on the Influence of Maternal Attachment Representations on Maternal Heart Rate Mediated by Perceived Stress

**DOI:** 10.3390/brainsci14050412

**Published:** 2024-04-23

**Authors:** Franziska Koehler-Dauner, Lena Peter, Eva Roder, Jörg M. Fegert, Ute Ziegenhain, Christiane Waller, Anna Buchheim

**Affiliations:** 1 Department of Child and Adolescent Psychiatry and Psychotherapy, University of Ulm, 89073 Ulm, Germany; lena.peter@uni-ulm.de (L.P.); joerg.fegert@uniklinik-ulm.de (J.M.F.); ute.ziegenhain@uniklinik-ulm.de (U.Z.); 2 Department of Psychosomatic Medicine and Psychotherapy, University Hospital of Ulm, 89081 Ulm, Germany; evi.roder@web.de (E.R.); christiane.waller@klinikum-nuernberg.de (C.W.); 3 German Center for Mental Health (DZPG), Partner Site Ulm, 89077 Ulm, Germany; 4 Department of Psychosomatic Medicine and Psychotherapy, Nuremberg General Hospital, Paracelsus Medical University, 90419 Nuremberg, Germany; 5 Institute of Psychology, University of Innsbruck, 6020 Innsbruck, Austria; anna.buchheim@uibk.ac.at

**Keywords:** attachment representation, perceived stress, heart rate, stress resilience

## Abstract

Past findings have suggested that there is a link between attachment representations and reactions towards stress (subjective and physiological). The aim of this study was to examine the mediating effect of perceived stress on the association between attachment representation and physiological changes, specifically heart rate. As part of a long-term study investigating the transgenerational transmission of childhood maltreatment, *n* = 163 mothers participated in multiple assessments. The Adult Attachment Projective Picture System (AAP) was used to measure maternal attachment representation, categorizing individuals as securely or insecurely attached. Perceived daily stress was assessed using the Perceived Stress Scale 14 (PSS-14), and maternal baseline heart rate (HR) was measured via electrocardiography during a laboratory visit. The results revealed that the representation of secure attachment had a significant reducing effect on both the mother’s perceived daily stress and heart rate. Furthermore, the association between secure attachment representation and heart rate was mediated by perceived stress. This study emphasizes the role of attachment representation in maternal well-being, highlighting its impact on stress and physiological responses.

## 1. Introduction

According to Bowlby’s attachment theory [1], there exists a biological-based system of behaviors which modulates proximity between an infant and its caregiver to protect the infant’s life from external threats. Several studies have demonstrated that adult attachment is connected to psychological and biological systems that govern the evaluation, reaction to, and recovery from attachment-related threats and stressors [1,2,3,4,5,6,7,8,9,10,11,12,13,14].

Research on the neuroscience of human attachment represents a broad spectrum of contemporary approaches to investigate biologically based systems that guide the cognitive and emotional processes of intimate and significant relationships. This spectrum includes studies and theoretical reviews that discuss neurobiological substrates (e.g., fMRI, EEG, psychophysiology, endocrine parameters, genetic polymorphisms) using a range of psychometric approaches to attachment assessment (interview, e.g., Adult Attachment Interview, AAI, [15]), the Adult Attachment Projective Picture System (AAP, [16]), and a self-report questionnaire (e.g., Relationship Scales Questionnaire [17]). In sum, this growing body of research has broadened our understanding of how attachment is related to impaired or balanced emotion regulation (see e.g., [13,18,19]).

In self-report studies, adults scoring high in attachment anxiety tend to be more sensitive to threats, leading to higher levels of perceived stress and an increased likelihood of ruminating over events [20]. Conversely, adults scoring high in attachment avoidance are more inclined to employ defensive strategies, primarily repression, to manage unpleasant and emotionally stressful situations [20]. Attachment style appears to be closely linked to interpersonal and intrapersonal factors, including stress and resilience [21]. Studies demonstrate that adults with a secure attachment style exhibit greater resilience to stress, while those reporting an insecure attachment style are more vulnerable to stress and tend to exhibit dysregulations [22,23,24,25]. Consistent with these findings, mothers with an insecure attachment style reported a greater lack of coping strategies and more severe depressive symptoms during the stressful period of the SARS-CoV-2 pandemic compared to mothers with a secure attachment style [26,27].

Attachment research has, in the past, focused mainly on subjective reports of stress and its symptoms, but recently, objective markers have also been increasingly studied [28,29,30,31,32]. It has been shown that stressful situations activate the attachment system [1,33], and physiological systems are the main indicators of stress responses [21]. Previous attachment research points to two relevant aspects: firstly, secure attachment has been identified as a critical buffer against physiological stress reactivity (e.g., [34]). These individuals use their attachment figures as a “haven of safety” and can express their feelings more confidently [21]. Secondly, in contrast, insecure attachment is associated with impaired emotion regulation [18,35], with individuals either exhibiting more feelings of anger and less autonomy (ambivalent/preoccupied) or deactivating their attachment distress (avoiding/dismissing), resulting in an increase in attachment-related physiological stress reactivity [11,18].

Maunder et al. [36] explored the impact of attachment security on subjective responses and heart rate variability during standardized stress, discovering that individuals with greater attachment security reported lower subjective stress levels. This finding is consistent with other research, indicating a detrimental effect of insecure attachment, particularly anxious attachment, on subjective stress [37,38,39,40]. They also identified an inverse relationship between an avoidant attachment style and high-frequency heart rate (HR). It is believed that immediate HR increases in response to stress enable enhanced oxygen and energy supply to manage stressful situations. Moreover, individuals’ attachment representations influence their stress responses, with secure attachment aiding better coping mechanisms and insecure attachment styles linked to heightened stress susceptibility [4,7,40]. The authors interpret their findings in terms of reduced neural control over visceral states associated with avoidant attachment [37,38,39,40]. In contrast, Diamond and colleagues [21] observed higher skin conductance levels in response to psychological stress among individuals with avoidant, but not anxious, attachment styles. However, a recent study investigating physiological responses to social stress found no differences in heart rate reactivity across different attachment styles [41]. Consequently, the precise nature of the relationship between physiological stress reactivity and attachment representation remains incompletely understood, and as far as we know, a mediation model incorporating an individual’s attachment representation, subjective stress, and heart rate response to stress has not yet been examined.

This study pursues the hypothesis that longitudinally perceived stress can influence the mean HR during an attachment-associated stress response. This consideration is based on the realization that stress is a complex, dynamic response of the organism that has not only immediate but also long-term effects on physiological processes. It is known that chronic or long-term stress can affect the regulatory system of the autonomic nervous system, which in turn controls the HR [21,41]. Assuming that longitudinal perceived stress is associated with increased strain over a prolonged period of time, it is plausible to assume that this continuous strain may affect mean HR during attachment-associated stress responses. Particularly in attachment response contexts, additional stresses and demands could amplify the physiological response [40]. The focus of this work is also on the HR during the stress paradigm, specifically to investigate a momentary state measurement of the stress response. The HR specifically during the Stranger Situation Procedure should thus provide a snapshot of the acute stress level or a direct insight into the physiological reactions to this specific attachment-associated stressor. This consideration emphasizes the importance of investigating not only the immediate effects of perceived stress but also the long-term effects on physiological dysregulation, particularly in the context of attachment representation.

**Figure 1:**
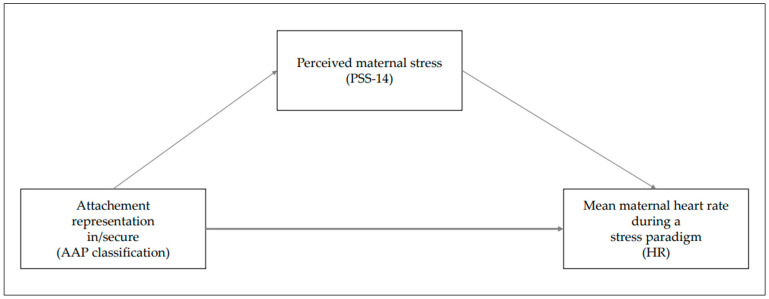
Mediation of attachment representation and mean maternal heart rate through perceived stress of the mother.

The aim of this study was to investigate the mediating effect of psychological variables, such as longitudinally perceived stress, between attachment representation and HR in mothers 12 months postpartum within a stress paradigm. Specifically, it can be hypothesized that the presentation of secure versus insecure attachment has a significant effect on maternal perceived daily stress in the past 12 months postpartum, that the presentation of secure versus insecure attachment has a significant effect on mean maternal HR under a stress paradigm, and that the relationship between the presentation of secure or insecure attachment and mean maternal HR is mediated by maternal perceived stress in the first year of the child’s life. The following model (Figure 1) was developed to be tested in the present study.

## 2. Materials and Methods

### 2.1. Study Design

The study TransGen is a research consortium focused on examining both protective and risk factors associated with the transgenerational transmission of childhood maltreatment (CM). This interdisciplinary effort integrates psychological, biological, and social perspectives within a prospective study design. The project received funding from the Federal Ministry of Education and Research from 2013 to 2016, with additional interim funding in 2017. Ethics approval was obtained from the Ethics Committee of Ulm University, and the research was conducted in adherence to the relevant guidelines and regulations. Data collection involved studying psychological, biological, and social factors in mothers and their newborn children through a prospective study design [13,42,43].

### 2.2. Participants

Between October 2013 and December 2015, healthy women who had given birth in the maternity ward of the Department of Obstetrics and Gynecology at Ulm University Hospital were recruited within 1–6 days after delivery of their healthy child. Recruitment excluded women under 18 years old, those with gestation of less than 37 weeks, those with insufficient proficiency in the German language, individuals with severe childbirth complications, health issues related to the mother or child’s general health or cardiology, current drug use, history of psychotic disorders, or recent infections. A total of 163 mothers provided their written informed consent. These mother–child pairs were then invited to attend five follow-up visits (t1, t2, t3, t4, and t5) consisting of laboratory and home visits when the children were 3 months old (t1), 12 months old (t2), 3 years old (t3), in preschool (t4), and in the first two years of primary school (t5). Maternal attachment representations were assessed only at t1. The analysis presented here is based on data collected at the first three measurements (t0, t1, t2) and includes data from participants in this study only. Attrition from t1 (*n* = 280) to t2 (*n* = 163) was due to personal reasons, lack of interest, time conflicts, and procedural errors during data collection. This accounts for the relatively small sample size of mothers who experienced childhood maltreatment (*n* = 72). A complete dataset including all measurements from t0 to t2 was required for the present analysis, resulting in a dataset of 163 mothers at t2. At t1, mothers’ ages ranged from 18 to 43 years with a mean age of 32.82 years (SD = 4.22 years). Participating mothers exhibited above-average levels of education, with 31.5% having achieved at least a bachelor’s degree.

### 2.3. Measures

#### 2.3.1. Maternal Attachment Representation

At T1, all mothers underwent assessment using the Adult Attachment Projective Picture System (AAP [16]), conducted by trained psychologists. The AAP [16,44] is a validated measure that evaluates adult attachment representation through the analysis of narrative responses to a series of seven drawings depicting attachment-related scenes such as solitude, illness, or separation. Each picture stimulus is coded for content and defensive processes, which guide the analysis of the narratives (for a more detailed description of the coding and classification procedure, see [16,45]).

The AAP classifies the four established attachment categories: secure, insecure—dismissing, insecure—preoccupied, and unresolved attachment. For our present study, the attachment representations of the mothers were divided into two major classifications *secure* and *insecure*. Insecure attachment includes dismissing and preoccupied classifications; here, any frightening or threatening material (e.g., desperately alone, death, attack, abuse, helplessness, danger, failed protection, isolation) that may appear in the story is contained (i.e., resolved). The insecure attachment group here also includes unresolved attachment, which refers to a group of individuals who are not able to regulate and contain or reorganize stories that evidence frightening or threatening material. In short, unresolved individuals are flooded and dysregulated by their attachment fears, which becomes evident in their disorganized narratives.

Studies demonstrate good psychometric properties of the AAP in adults [16,46,47] and adolescents [48] by showing high inter-rater reliability [44,46,47], discriminant validity in controls and clinical patients, and test–retest reliability [16,41]. Also, a high concurrent validity with the *Adult Attachment Interview* (AAI; Main and Goldwyn, 1998) was demonstrated in several independent samples [16,34].

Maternal AAP classification was performed by two independent certified judges. Inter-rater reliability showed significant concordance for the four-group classification (*κ* = 0.95, 95% confidence interval [0.88, 1.04], *p* < 0.001), and for the two-group classification (secure vs. insecure, *κ* = 0.96), 95% confidence interval [0.91, 1.00], *p* < 0.001.

#### 2.3.2. Maternal Perceived Everyday Stress

Mothers’ perceived daily stress levels were assessed starting from the birth of the child at time t0, with additional measurements at times t1 and t2, using the Perceived Stress Scale 14 [49]. The PSS14 is a commonly used and well-established self-report scale that measures perceived stress on a 5-point scale, comprising seven negative and seven positive items. Higher scores indicate higher levels of perceived stress. To derive an overall measure of mothers’ perceived everyday stress, the z-standardized values from all three measurement time points were summed. This stress measure represents the longitudinally recorded daily stress level of the mother, beginning after childbirth and continuing until the child reaches preschool age.

#### 2.3.3. Maternal Heart Rate

The baseline heart rate of the mothers was measured during the strange situation procedure (SSP) at time t2 using wireless lightweight mobile units (Mindware Technologies, Hahanna, OH, USA). For this purpose, seven disposable spot electrodes were placed on the mothers’ skin.

Before analyzing, the ANS data were filtered and scored using the mindware software (BioLab 3.1 1.0J; Mindware Technologies, Gahanna, OH, USA). Artifacts derived from child’s movements, speech, or close physical contacts were eliminated. Every segment of the data was checked and corrected for inaccurate R-peak detections by trained coders. Each of the 8 episodes (baseline period (e1) and 7 subsequent episodes (e2–e8) of the SSP) of the SSP were divided into 30 s segments. Only the first six segments of each episode were used for statistical analysis. In cases where fewer than six segments were available, all the available data were used. Data cleaning procedures, including random monitoring, were based on previously described methods [18].

In this study, only the mean heart rate, which is the average of the heart rate over the entire stranger situation, was used. The decision to use the mean heart rate was made to provide a comprehensive insight into the physiological response of the mothers during the entire stranger situation test, which, in this case, acted as a simulation of attachment-associated stress. This made it possible to look not only at relative stress at a particular moment but also to capture the cumulative response over the entire duration of the test sequence and, in particular, to obtain a complete picture of stress-induced changes in heart rate. The use of mean heart rate thus provided a more comprehensive basis for analyzing the relationship between maternal attachment representation, longitudinally perceived stress, and a physiological response change in this study.

### 2.4. Statistical Analysis

Data were analyzed using SPSS Statistics 28.0 (IBM Corp., 2021, Armonk, NY, USA), and the significance level was set at 0.05 as the critical alpha level. Descriptive statistics with means, standard deviations, and relative frequencies are reported. Descriptive statistics and bidirectional Pearson correlations of model and control variables were calculated before testing the hypotheses. The model variables were attachment representation using the AAP, maternal longitudinal perceptual stress collected by measuring the PSS-14 at four measurement time points, and maternal heart rate. Most of the available data met all the statistical requirements, which meant that the data were checked with independent t-tests and a mediation analysis using the PROCESS macro for SPSS. This made it possible, on the one hand, to compare the groups in terms of maternal HR and perceived stress using independent t-tests and, on the other hand, to examine the mediation relationships between group membership in the securely or insecurely attached group and maternal heart rate mediated by perceived daily stress. The PROCESS macro can be used in particular to investigate mediation hypotheses, as PROCESS performs regression-based path analyses according to the least squares method. Using the PROCESS macro for SPSS and Model 4, 5000 bootstrap estimates were run along with heteroskedasticity-consistent standard errors to generate 95% bias-corrected confidence intervals for the observed indirect effects. In addition, all the analyses have been controlled for age and education level as well as mean actual social support (PSSQ) collected at earlier measurement time points.

Prior to the study, mothers were divided into two groups based on the attachment representation using the AAP. In the present study, mothers’ attachment representations were divided into two main categories: secure (F) and insecure (insecure—dismissing (Ds), insecure—-preoccupied (E), and unresolved attachment (U)), as the sample size in each category would otherwise be too small. Some of the analyses were subjected to the Greenhouse–Geisser correction procedure.

## 3. Results

### 3.1. Descriptive Analysis

Data from *n* = 163 mothers were used in this study. The mean age of the mothers at t1 was M = 32.8 years (SD = 4.2), with a range of 18 to 43 years. In total, 59.9% of the women had a university degree, 15 % had a high school degree, 18% had a secondary school degree, and 6.9% had a lower secondary school degree. Only 0.2% reported that they had no high school diploma. On average, mothers had a mean actual social support score (PSSQ) of 4.0 (SD = 0.8), with a range of 2.2 to 6.0.

Examination of the descriptive statistics revealed that 70% of the mothers in this sample had insecure attachment representations. The mean perception of stress (PSS-14) over the three measurement points averaged M = 22.2 (SD = 8.2), with a minimum of 8.18 and a maximum of 66.9. The mean heart rate of the mothers during stress induction ranged from 53.0 to 115.8, with an average of M = 78.2 (SD = 12.6) (see Table 1).

### 3.2. Correlation Analysis

Attachment representation correlated with mean maternal heart rate and mean perceived stress. Likewise, a correlation between mean longitudinal perceived stress and mean heart rate was demonstrated (see Table 1.).

**Table 1:** Correlation table: correlations between attachment representation, perceived stress, and HR.

Variable	Mean Value	Standard Deviation	Insecure/ Secure Attachment	Perceived Stress
Insecure/ secure attachment	1.7	0.459		
Perceived stress	22.22	8.66	0.541 **	
Maternal HR	78	13	0.822 **	0.534 **

** The correlation is significant at the 0.01 level (2-sided), *p* < 0.001.

### 3.3. Mediation

A t-test for independent samples was used to examine whether there was a correlation between the attachment representation groups and perceived stress. The results show that the group differences are significant in terms of perceived stress. According to Cohen’s d, there is a strong negative correlation. To investigate whether there was a correlation between the group differences and maternal heart rate, a t-test for independent samples was also calculated. The results show that the differences between secure and insecure attachment representations also become significant in relation to mean maternal heart rate. According to Cohen’s d, there is a negative correlation.

**Figure 2:**
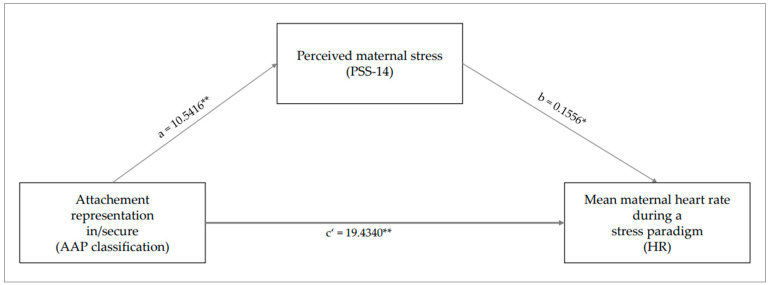
Results of the mediation of attachment representation and mean maternal heart rate through perceived stress of the mother. * The path is significant at the 0.05 level, *p* < 0.05. ** The path is significant at the 0.01 level, *p* < 0.001.

A mediation was also calculated for the variables mentioned using PROCESS. It was found that there was a direct correlation between the attachment representation groups and maternal heart rate, which was mediated by the mother’s perceived stress. The calculation of the direct effect (c’) of the model showed that attachment representation had a significant effect on maternal heart rate: B = 19.434, Bse = 1.360, t = 14.289, *p* = 0.00034. The mediation model also showed a significant effect, so the associations between the attachment groups and the mother’s perceived stress, as well as between the perceived stress and the maternal heart rate, became significant. The overall moderated mediation model was measured by the index of moderated mediation: index = 1.610 (95% CI = 0.301; 3.088). Since the value zero is not within the confidence interval, it can be assumed that there is a significant mediation effect. Thus, the mediation model can be confirmed by the available data (see Figure 2).

## 4. Discussion

The present study aimed to investigate the relationship between maternal attachment representation, perceived daily stress, and average heart rate (HR) under an attachment-associated stress paradigm. Our results show that mothers with an insecure attachment representation had significantly more perceived stress and a higher average heart rate compared to mothers with a secure attachment representation. Furthermore, the relationship between attachment representation and maternal heart rate was confirmed to be significantly mediated by the mother’s perceived stress. This confirmation of the proposed mediation model represents an important contribution to the current state of research; as far as we know, this mediation model has not yet been tested. In addition, our study revealed a possible mechanism of interaction between attachment representation and psychological and physiological processes, as discussed, but not shown, by Maunder et al. [35].

The present study contributes to the existing research by demonstrating a significant association between maternal attachment representations, perceived stress, and physiological responses. Our findings support previous studies that have shown that insecure attachment representations are associated with an increased experience of stress and an increased physiological stress response. One possible explanatory mechanism for these associations could be the interaction between psychological and physiological processes. Our findings suggest that a mother’s perceived stress response plays a mediating role in the relationship between attachment representation and physiological response, suggesting that psychological factors such as attachment representation may influence the physiological regulation of the stress system.

The confirmation of the mediation model emphasizes the importance of the consideration of the psychological dimensions in the study of physiological responses and contributes to expanding upon the understanding of the complexity of the relationship between attachment, stress, and health. Furthermore, our results suggest that interventions to improve maternal attachment security could positively influence not only mothers’ psychological well-being but also their physiological responses. By uncovering this mechanism of interaction between psychological and physiological processes, we contribute to the development of targeted interventions that can promote both attachment security and health in mothers and their children. Overall, our results highlight the relevance of considering attachment representation when studying stress and physiological responses and provide an important impetus for future research and intervention strategies in the area of maternal health and attachment. 

### Strengths and Limitations

Our study has several strengths that underscore its importance. First, the careful measurement of both maternal attachment representation and physiological parameters was performed, ensuring the accuracy and reliability of the data. Second, a mediation model was used to examine the relationship between attachment representation, perceived stress, and physiological response. This methodological approach allowed for a detailed analysis of the underlying mechanisms. Third, confirming the mediation model contributes to expanding existing knowledge, as previous studies have rarely considered the mediated influence of perceived stress. Finally, our results highlight the clinical relevance of maternal attachment representation and its impact on stress and physiological responses, providing an important impetus for future interventions.

There are several limitations to consider: First, it should be noted that the sample was not representative despite all efforts. For example, the subjects had an above-average level of education and socioeconomic status. These factors may act as protective factors for mental illness and stress and may therefore have influenced the results. Furthermore, studies show that higher education and socioeconomic status are positively related to psychological well-being and life satisfaction. Furthermore, there is evidence that women who were victims of harassment with lower levels of education had more severe trauma-related symptoms than women with higher levels of education. This finding suggests that post-traumatic vulnerability can be mitigated by educational level [50]. Apart from the higher-than-average level of education and socioeconomic status, there are other characteristics of the present sample that may have skewed the results. For example, the majority of mothers in our sample lived in a partnership. It is possible that the high proportion of those in a partnership in our sample reduced the effect of single parents experiencing a lack of social support.

Secondly, in terms of attachment representation, this study only compared secure and insecure attachment representations due to the sample size. This dichotomization of the AAP classification may also influence the results, as only one insecure group consisting of three different attachment patterns was contrasted with one group of secure attachment representation.

In addition, a limitation of this study was data collection problems due to infant movement and physical contact between mother and infant, followed by electrode interference. In addition, there were data collection failures due to procedural errors. This explains the comparatively small sample size of mothers without the experience of childhood maltreatment experience with a complete dataset (*n* = 91).

Accordingly, subsequent studies in this research area should take the following things into account. For example, an attempt should be made to obtain a more representative sample of women from all socioeconomic status groups. Furthermore, if possible, a different classification of groups should be chosen. Based on this, a comparison of different mediation models with subdivisions of different degrees of severity of insecurity in the attachment representation would also be conceivable and interesting for future studies.

## 5. Conclusions

In conclusion, this study elucidates the impact of attachment representation on stress and maternal heart rate. The findings emphasize the importance of secure attachment representation in lowering stress levels and shaping physiological responses to stressors. Moreover, the mediating role of perceived stress in the relationship between attachment representation and heart rate creates an understanding about the complex interplay between psychological and physiological factors. By providing insights on the impact of attachment on stress and physiological responses, this study provides valuable implications for clinical interventions and preventive strategies aimed at promoting maternal well-being.

## Data Availability

The raw data supporting the conclusions of this article were made available by the authors on reasonable request. The data are not publicly available, as the study is still ongoing and has not been completed.
